# Wild snapdragon plant pedigree sheds light on limited connectivity enhanced by higher migrant reproductive success in a fragmented landscape

**DOI:** 10.12688/openreseurope.14281.1

**Published:** 2021-12-01

**Authors:** Laura Gervais, Pierick Mouginot, Anais Gibert, Oceane Salles, Mathieu Latutrie, Jesaelle Piquet, Juliette Archambeau, Benoit Pujol

**Affiliations:** 1 CRIOBE PSL Université Paris : EHPE-UPVD-CNRS, Université de Perpignan, USR 3278, CNRS, Perpignan, France; 2 BIOGECO UMR 1202, INRAe, Pessac, France

**Keywords:** Functional connectivity, dispersal, wild population pedigree, fragmented landscape

## Abstract

**Background:** In contrast with historical knowledge, a recent view posits that a non-negligible proportion of populations might respond positively to habitat fragmentation. Populations might thrive in a fragmented landscape if functional connectivity, i.e., the net flow of individuals or their genes moving among suitable habitat patches, is not restricted. Alternatively, functional connectivity might be typically limited but enhanced by a higher reproductive success of migrants.

**Methods:** We tested for this hypothesis in wild snapdragon plants inhabiting six patches separated by seawater in a fragmented Mediterranean scrubland landscape. We reconstructed their pedigree by using a parentage assignment method based on microsatellite genetic markers. We then estimated functional connectivity and the reproductive success of plants resulting from between-patch dispersal events.

**Results:** We found that wild snapdragon plants thrived in this fragmented landscape, although functional connectivity between habitat patches was weak (i.e. 2.9%). The progeny resulting from between-patch dispersal events had a higher reproductive success than residents.

**Conclusion:** Our findings expose a remarkable aspect of fragmented landscapes, where weak functional connectivity was enhanced by higher reproductive success after migration. This process might have the potential to compensate at least partly the negative impact of fragmentation.

## Introduction

Human-induced or natural habitat fragmentation has long been assumed to adversely affect population persistence and distribution ( Fahrig & Merriam, 1994). In a fragmented habitat, theory predicts that the limited movement of individuals or limited gene exchanges among habitats enhances reproductive isolation and opens the way to genetic drift and loss of genetic diversity in small populations ( Aguilar *et al*., 2008; Gittleman *et al*., 2000; Vranckx *et al*., 2012). As a consequence, the long-term survival and adaptive potential of populations was historically expected to decline in a fragmented habitat ( Frankham, 1995; Wade *et al*., 2017). However, a recent meta-analysis demonstrated that a non-negligible part of populations was not affected by or responded positively to habitat fragmentation ( Fahrig, 2017). For example, a higher abundance of sawtooth grain beetle and a higher species richness in ant communities were found in fragmented rather than continuous habitats ( Bancroft & Turchin, 2003; Dauber *et al*., 2006). There is accruing evidence that populations are not negatively impacted, and may even benefit, from fragmented habitat, but the underlying mechanisms remain poorly understood ( Fahrig, 2017; Fahrig *et al*., 2019).

Maintenance of high functional connectivity (i.e., the net flow of organisms and their genes moving among suitable habitat patches, Tischendorf & Fahrig, 2000) counteracting reproductive isolation might explain why some populations thrive in a fragmented habitat ( Dick *et al*., 2003; Nason & Hamrick, 1997). To date, functional connectivity has been widely evaluated by using estimates of dispersal rates and genetic differentiation ( Auffret *et al*., 2017). The flow of individuals or genes can only counteract reproductive isolation in the long term if migrant individuals successfully reproduce and migrant genes are successfully transmitted to the next generations (a mechanism known as effective connectivity; Cayuela *et al*., 2018; Robertson *et al*., 2018). For example, in snail kite populations characterised by a high dispersal rate, and therefore high functional connectivity, effective connectivity was limited because migrants had a low reproductive success ( Robertson *et al*., 2018). Conversely, populations characterised by limited dispersal rates but thriving in a fragmented habitat might have maintained effective connectivity through high migrant reproductive success ( Lowe & Allendorf, 2010). This latter hypothesis remains poorly tested, as few studies jointly assessed connectivity and migrant reproductive success in the wild (but see Robertson *et al*., 2018; Robertson *et al*., 2019; Vasudev & Fletcher, 2016).

In plants, functional connectivity involves the dispersal of seed and pollen, resulting in the establishment of a new adult plant (i.e., effective dispersal, Auffret *et al*., 2017; Schupp *et al*., 2010). Despite the use of parentage assignment in plant functional connectivity studies ( Kamm *et al*., 2010; Moran & Clark, 2011; Sork *et al*., 1999), which offers the opportunity to assess the reproductive success of these new plants, their reproductive success has received little attention ( Aguilar *et al*., 2019; Auffret *et al*., 2017). One reason for this is the need for long-term studies. Here, we reconstructed a ten-year multigenerational pedigree of wild snapdragon plants ( *Antirrhinum majus* L.) thriving in a fragmented landscape in southern France. These snapdragon plants inhabit patches of suitable Mediterranean scrubland isolated for a few hundred meters by seawater, which corresponds to the remains of a site where salt was manufactured by exploiting semi-natural crystallisation ponds. Here we assessed jointly functional connectivity between patches and the reproductive success of plants.

Seed dispersal by small animals and insects can generally reach ca. hundred meters ( Uroy *et al*., 2019) and up to several kilometers when large or migratory animals are involved ( Mueller *et al*., 2014; Vittoz & Engler, 2007). In snapdragon plants, seed dispersal occurs by gravity and should therefore be geographically-limited. We therefore did not expect seed dispersal to connect patches of land separated by seawater. Pollen dispersal might enable connectivity because snapdragon plants are pollinated by bumblebees, carpenter bees and other large-sized pollinators known to fly distances larger than the distance separating patches in this fragmented landscape ( Chapman *et al*., 2003). However, the distance covered by these pollinators is potentially limited in a fragmented landscape, even at an extremely small spatial scale, e.g., across distances of ca. 40m ( Goverde *et al*., 2002). Although landscape fragmentation may affect forests at the sale of kilometers, it might affect insect pollinated plants at the scale of a few hundred meters. We therefore expect to find typically limited functional connectivity and possibly genetic differentiation between these patches snapdragon plants thriving in a fragmented landscape. We tested for the rarely explored hypothesis that connectivity would be effective on the long term; in other words, that the progeny resulting from migration between patches would successfully reproduce.

## Results

### Demographic expansion of snapdragon plants in a fragmented landscape

Over ten years (2010–2019), we sampled ca. twelve thousand flowering plants (N=12594) on six patches (from 2508 to 12547m ^2^ area) isolated from each other by a few hundred meters of seawater (from 158 to 1627m, Table 1). Our long-term survey data showed that these snapdragons are ongoing a demographic expansion. The population size has increased tenfold in ten years, with an average annual population growth rate (λ) ranging from 1.25 for the easternmost patch (Patch 6) to 2.04 for the westernmost patch (Patch 1) ( Table 2).

**Table 1.** Mean distance between and within patches in meters. On the diagonal is the mean distance between individuals within a patch, in meters. Off-diagonal is the mean distance between individuals of the different patches, in meters.

	Patch 1	Patch 2	Patch 3	Patch 4	Patch 5	Patch 6
Patch 1	94.8					
Patch 2	579.9	47.6				
Patch 3	947.9	407.5	23.5			
Patch 4	1177.7	630.8	229.9	40.5		
Patch 5	1570	1014.9	622.1	392.6	46.2	
Patch 6	1627.9	1057	690.1	472	158.7	20.4

**Table 2.** Growth rates per site and year. Population growth rates >1 (reflecting expansion) are in bold. If there was no individual sampled in a patch on a given year, the growth rate of the subsequent year could not be calculated. In this case, we reported the number of plants, indicated in italics between brackets.

	patch					
growth rate	1	2	3	4	5	6
Median	2.04	1.40	1.33	1.46	1.75	1.25
2011	11	43	[1]	[28]	3.17	1.4
2012	0	1.4	8	1.36	0.08	0.57
2013	[161]	6.72	7.5	2.53	14.5	5.5
2014	0.04	0.22	0.13	0.58	1.11	0.22
2015	78	4.69	14.88	1.46	3.46	5.05
2016	0.92	2.12	2.16	4.26	1.86	2.33
2017	2.04	0.6	0.32	0.21	0.7	0.49
2018	0.18	0.64	0.98	0.73	1.75	1.12
2019	3.02	1.23	1.33	2.04	0.91	1.25

### High genetic diversity and low genetic differentiation

**Figure 1.**
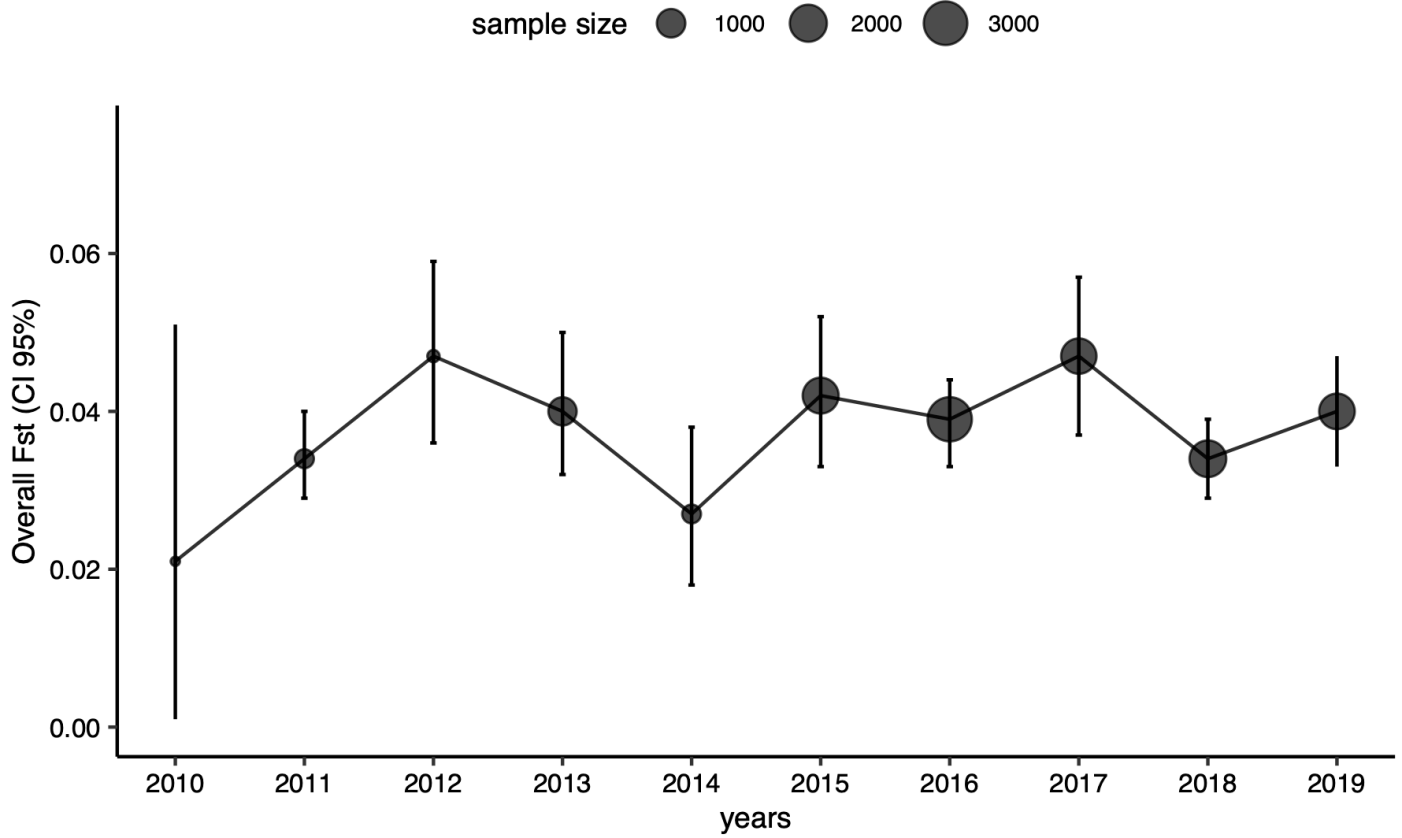
Genetic differentiation (multilocus Fst) amongst patches over 10 years. Dots and bars around the dots correspond respectively to global Fst estimates measured amongst patches and their associated 95% confidence intervals. Dot size is proportional to the sample size used to estimate Fst.

Genetic diversity (estimated by Nei’s expected heterozygosity) was high in every patch of snapdragon plants in this fragmented landscape ranging from 0.678 for patch 4 to 0.707 for patch 5 (average Hs=0.69 ± 0.004). These diversity values are similar to those previously found in populations distributed across the species geographic range (average Hs=0.65±0,02; Pujol *et al*., 2017). Other genetic parameters (e.g., allelic richness) corroborating this high diversity can be found in Table 3). We also found weak but significantly different from zero genetic differentiation amongst patches (Fst=0.04, p=0.001). Fst between pairs of patches ranged from 0.014 to 0.079 (p<0.001 for all pairwise Fst estimates; Table 4). We did not observe any identifiable trend in time or space shaping Fst ( Figure 1).

**Table 3.** Descriptive statistics for microsatellites markers. It includes the name of the locus (Locus), allelic richness (A), number of plants genotyped at a given marker (N), observed heterozygosity (Hobs), expected heterozygosity (Hexp), polymorphic information content (PIC), Parent-pair exclusion probability (PPexp).

Locus	A	N	Hobs	Hexp	PIC	PPexp
Antibg36	17	12486	0.848	0.880	0.868	0.910
Antibg38	6	12541	0.594	0.608	0.533	0.488
Antibg23	7	12539	0.546	0.579	0.545	0.558
Antibg40	3	12546	0.642	0.658	0.584	0.513
Antibg11	19	12526	0.831	0.829	0.811	0.853
Antibg18	6	12542	0.587	0.604	0.559	0.552
Antibg03	17	12512	0.633	0.659	0.627	0.656
Antibg02	10	12516	0.773	0.809	0.785	0.815
Antibg10	29	12317	0.642	0.842	0.824	0.864
Antibg12	10	12550	0.653	0.65	0.597	0.564
Antibg20	9	12494	0.691	0.723	0.80	0.687
Antibg30	29	12470	0.875	0.925	0.920	0.961
Antibg29	7	12545	0.564	0.576	0.494	0.436
Antibg33	24	12511	0.827	0.888	0.878	0.926
Antibg39	9	12536	0.576	0.598	0.569	0.591
Antibg22	6	12517	0.691	0.742	0.699	0.692
Antibg14	17	12503	0.855	0.893	0.884	0.928
Antibg27	6	12557	0.457	0.461	0.385	0.326

**Table 4.** Pairwise Fst values between patches estimated in 2019. All p-values are <0.0001.

Patch	1	2	3	4	5
2	0.042	-			
3	0.079	0.049	-		
4	0.067	0.043	0.015	-	
5	0.048	0.035	0.035	0.022	-
6	0.064	0.048	0.034	0.029	0.014

### Conservative parentage assignment and missing parentage links

**Figure 2.**
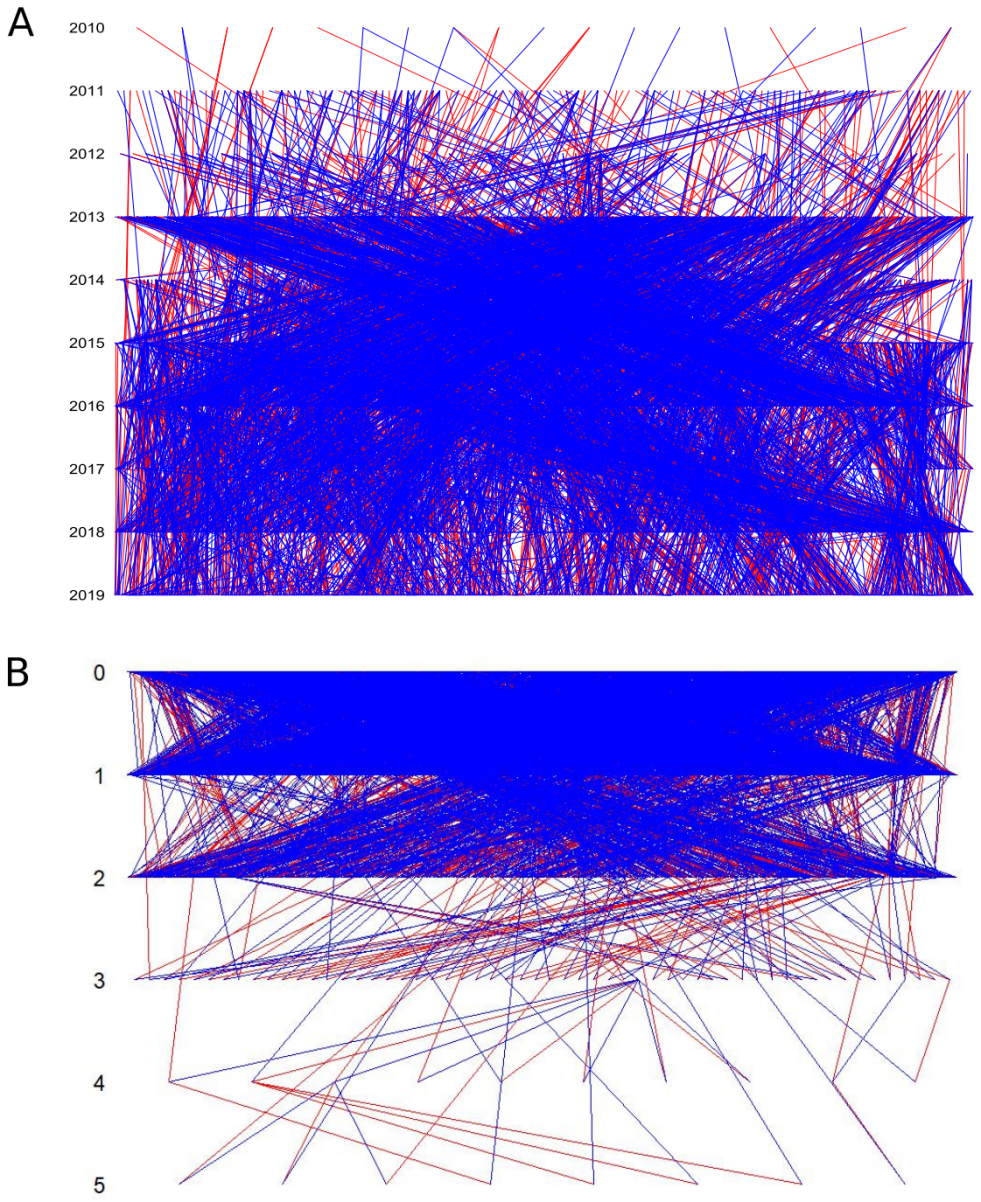
Pedigree represented per year ( **A**) and by generations ( **B**). Each line represents a parent-offspring link. Red and blue lines represent the closest and the farthest parents, respectively.

The long-term survey of snapdragon plants allowed us to build a multigenerational pedigree including ca. 40% of the genotyped plants (N=5053; Figure 2). For 23% of the genotyped plants, both parents were assigned (n=2818 parent-offspring triads). We used greatly effective markers to reconstruct the pedigree (cumulated markers probability to exclude a ‘random’ individual from parentage, Wang, 2007, PPexp>0.999, see Table 3 for more details). Our assignment approach was highly selective. We only included parents with high assignment probability (above 95%) in the pedigree. Although this final pedigree is highly conservative, it comprises a large number of plants and family links; 5053 individuals with 2235 founders, 2818 offspring with two identified parents (for which 2815 have spatial coordinates), 805 offspring with only one identified parent, 2571 parents, 420 full-sibling links, 17170 half-sibling links (see Table 5 and Figure 2). These plants are grouped in 234 families spanning across one to five generations ( Figure 2B) and composed on average by 21.5 individuals. The non-negligible part of plants with only one or no assigned parents was likely mothered or fathered by plants that we did not sample. Plants measured at the beginning of the survey probably had parents from before the sampling campaign (e.g., 94 % of unassigned parents in 2012 against 57% in 2019). In addition, some parents were likely missed in the surveyed area during fieldwork even if we conducted a thorough search of sexually mature plants in the area. Some unidentified parents might also likely be located outside the surveyed area. The presence of plants with at least one unidentified parent suggests migration from outside the studied area ( Bacles *et al*., 2006; Sebbenn *et al*., 2011).

**Table 5.** Pedigree description. The final pedigree is only composed of parent-offspring triads.

	Quantity
Number of individuals in the pedigree	5053
Parent-offspring links	5636
Full sibling links	420
Half-sibling links	17170
Grand-parent offspring links	1362
Full avuncular links	86
Full first cousin links	56
Half avuncular links	4596
Number of founders	2235
Maximum pedigree depth (in generations)	5

### Low functional connectivity between the six patches

**Figure 3.**
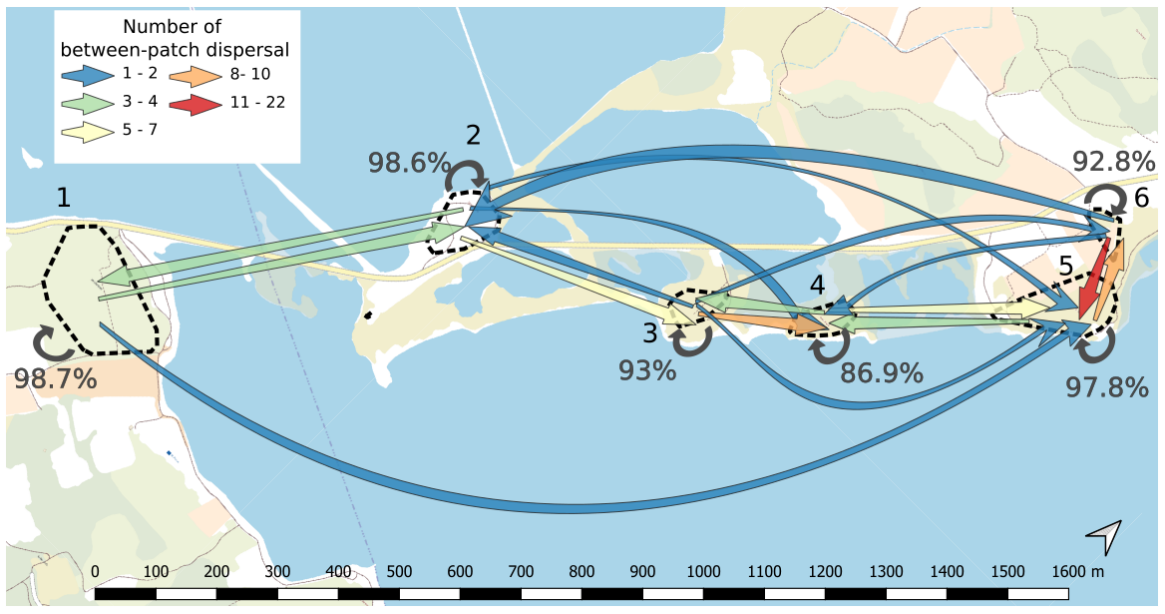
Connectivity map between patches. Each patch is delimited by dashed line and its corresponding number going from 1 to 6. Arrows indicate the presence of effective pollen dispersal from a given patch to another. The number of between-patch dispersal events ranges from 1 to 22 events and the colour of arrows represents this number (from blue to red, see legend on the figure). Circular arrows on each patch represent self-recruitment and are accompanied by the corresponding percentage. Background map is provided freely by IGN at https://www.geoportail.gouv.fr/donnees/plan-ign-v2

**Figure 4.**
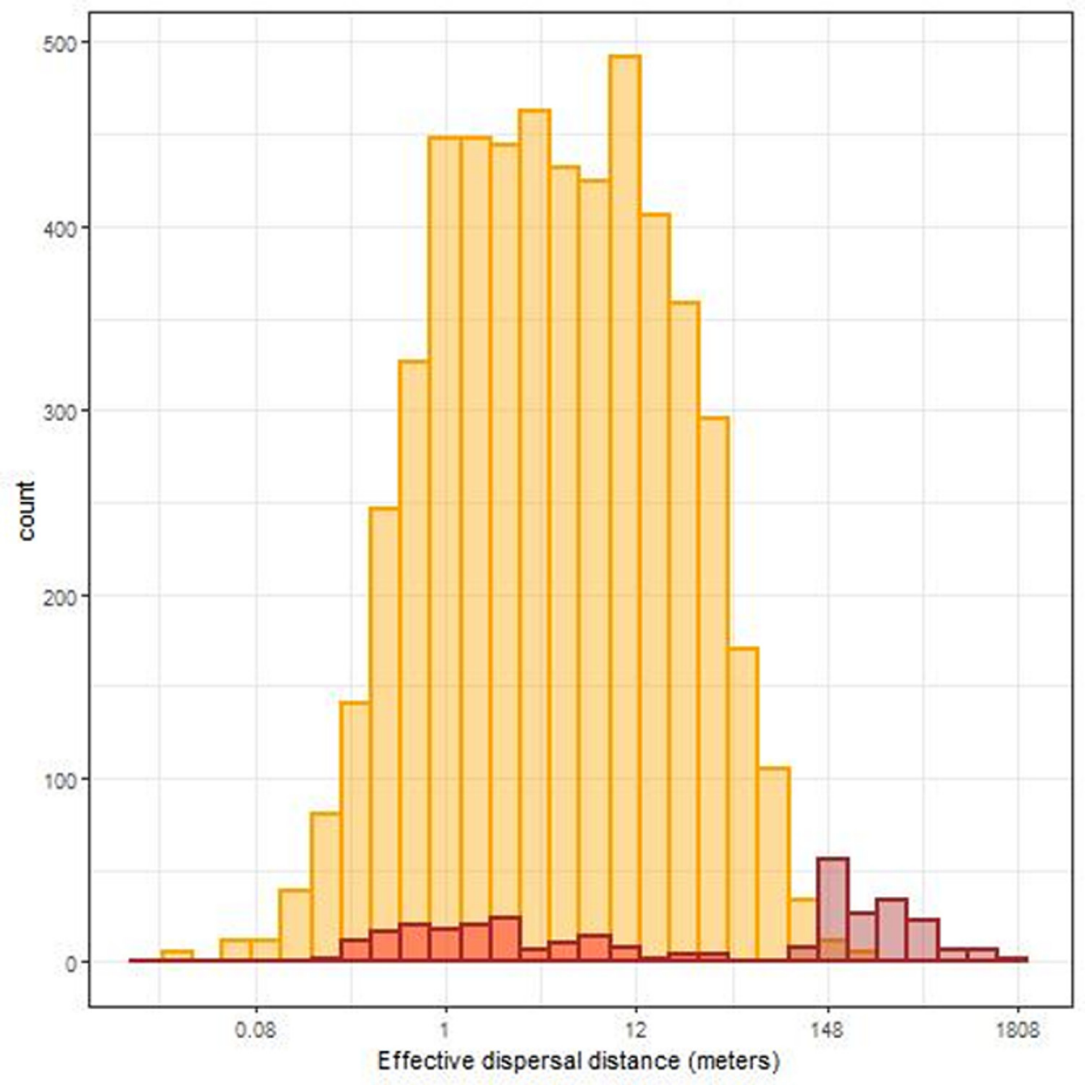
Distributions of the effective dispersal distances. Yellow bars represent dispersal distance between parent-offspring triads inhabiting the same patch. Bars with brown borders represents distance between parent-offspring triads, with one parent inhabiting on a different patch. Brown-bordered bars filled in red represent the distance between the closest parents and the offspring which is more likely representing seed dispersal, while brown-bordered bars filled in brown represent the distance between the farthest parents and the offspring.

Functional connectivity was estimated by the rate of between-patch effective dispersal events ( Auffret *et al*., 2017). The use of the multigenerational pedigree in combination with the spatial coordinates of the plants revealed that only 2.9% of offspring had one parent on a different patch (n=81 out of a total of 2818 offspring with two known parents, details per patch in Figure 3 and Table 6). This small proportion likely indicates low functional connectivity through effective pollen dispersal events among patches. On average, the pollen dispersal distance between patches was 345m (ranging from 99 to 1656m). In addition, we assumed that dispersal events between patches allowed us to identify the maternal parent located on the same site as the offspring. We estimated an average seed dispersal distance of 3.86m (ranging from 0.22 to 31.28m, Figure 4). Dispersal distance within patches ranged from 0.02 to 256.17m, including seed and pollen. Dispersal distances between patches were not necessarily higher than within patches (e.g., larger distances are found within patch 5 than between patch 5 and 6 or 3 and 4, Table 1). Yet only 37 dispersal events within patches out of 5359 (0.7%) occurred at a similar spatial scale than between-patch dispersal events ( Figure 4).

**Table 6.** Functional connectivity per patch. Outbound connectivity is the percentage of offspring on a different patch with the farthest parent on a given focal patch. Inbound connectivity is the percentage of offspring on the given focal patch with the farthest parent on a different patch. Total connectivity is the sum of inbound and outbound connectivity.

Patch	1	2	3	4	5	6
Outbound connectivity	1.7%	2.3%	8.6%	8.1%	1%	16%
Inbound connectivity	1.3%	1.4%	6.9%	13%	2%	7.2%
Total connectivity	2.95%	3.69%	14.45%	19.29%	2.98%	20.86%

### Little spatial and temporal variation in connectivity

Functional connectivity varied both spatially and temporally, but to a low extent. It increased from 2.8% in 2014 to 4.5% in 2016 and finally decreased to 1.4% in 2019.

At the spatial level, it decreased with the between-patch geographical distance (rho=-0.75, p=0.001), ranging from 0% between several pairs of patches separated by distance ranging from 472m to 1627.9m; to 1.9% between patches 5 and 6 separated by 158.7m ( Figure 3).

### Effective connectivity

Functional connectivity between patches was effective. We found that 64% of plants resulting from between-patch dispersal events successfully reproduced over the duration of the survey. Plants resulting from between-patch dispersal events had on average 0.79 offspring per plant. These plants had around twice as many offspring as plants with two resident parents that had on average 0.43 offspring (regression model parameter back-transformed estimate: 2.151, CI95%: 1.05-4.41, p=0.036).

## Discussion

Our survey revealed that thousands of snapdragon plants were thriving on patches of Mediterranean scrubland interrupted by seawater ponds formerly exploited for salt production. We found that these plants were undergoing demographic growth and characterized by high levels of genetic diversity that were similar to those of continuous populations at similar spatial scales ( Pujol *et al*., 2017; Ringbauer *et al*., 2018). The good condition of these snapdragon plants makes it an interesting system for investigating the mechanisms by which populations are not adversely affected in a fragmented landscape. The very weak but statistically significant genetic differentiation between patches, which illustrates the equilibrium of evolutionary forces that are migration and genetic drift, provided an ambiguous message about their potential actual reproductive isolation. For example, this result could be explained by recent connectivity following a past divergence in the process of being resolved but other scenarios might also explain this result. We used a connectivity approach based on the reconstruction of a multigenerational pedigree to clarify whether these snapdragon plants were reproductively isolated in this fragmented landscape.

### Low functional connectivity across the fragmented landscape

Our results showed that only 2.9% of offspring resulted from dispersal events between patches of snapdragon plants. Similarly low percentages indicating limited functional connectivity have previously been found in other species inhabiting fragmented landscapes ( Mueller *et al*., 2014; Sebbenn *et al*., 2011). Higher connectivity – up to an order of magnitude greater – has also been found in other species inhabiting fragmented landscapes ( Bacles & Ennos, 2008; Hebel *et al*., 2007; Wang *et al*., 2010). It is therefore legitimate to consider that functional connectivity between snapdragon plant patches was limited in this fragmented Mediterranean scrubland landscape where ecological barriers such as the seawater ponds separating the patches might have limited the movement of pollinators. Bumblebees and carpenter bees are the main pollinators of snapdragon plants ( Tastard *et al*., 2012). They are known to cover long distances ( Chapman *et al*., 2003). For instance, we detected pollen dispersal events between patches over distances up to 1656 meters. However, pollinators able to cover long distances rarely do so frequently ( Walther-Hellwig & Frankl, 2000; Zurbuchen *et al*., 2010). Pollination might have typically been favoured between neighbouring plants ( Kunin, 1997; Tastard *et al*., 2012). Our findings therefore did not support one of the possible explanations put forward for populations thriving in fragmented landscapes, namely the presence of high functional connectivity ( Fahrig, 2017; Fahrig *et al*., 2019).

### Higher reproductive success associated with connectivity

Reconstructing the multigenerational pedigree of wild populations is challenging but rewarding because it also provides information about the reproductive success of plants. Our results showed that plants resulting from between-patch dispersal events had higher reproductive success than plants with resident parents. Organisms colonising new habitats (e.g., geographic range expansion, biological invasion) are expected to have a higher fitness than the average fitness of resident organisms ( Bonte *et al*., 2014; Ronce, 2007). Organisms inhabiting fragmented landscapes can also be selected for their potential to invade other patches ( Williams *et al*., 2019). Our findings highlight the knowledge gained by evaluating offspring quality beyond the count of their number ( Aguilar *et al*., 2019). Although functional connectivity was low, migrant genes from other patches were therefore successfully integrated into the resident gene pool. Our findings contrast with a study in endangered birds where functional connectivity remained high in the fragmented landscape but the reproductive success was low, which was detrimental to the species ( Robertson *et al*., 2018; Robertson *et al*., 2019). Our approach in the wild cannot identify the mechanisms underlying the higher reproductive success of snapdragon plants resulting from between-patch dispersal events. However, it exposed a remarkable aspect of fragmented landscapes, where low functional connectivity is insured to be effective by a high migrant reproductive success, which may have the potential to compensate at least partly the negative impact of reproductive isolation.

## Conclusion

Our findings in snapdragon plants add up to the recent awareness that some species have the potential to thrive in a fragmented landscape. Our findings support an often-neglected hypothesis whereby typical weak connectivity in a fragmented landscape can be enhanced by a higher reproductive success of migrants. The extent to which migrants successfully transmit their genes to the next generations is rarely evaluated ( Aguilar *et al*., 2019; Robertson *et al*., 2018). Our findings highlight the benefit of integrating the reproductive success of migrants in studies evaluating connectivity in a fragmented landscape.

## Methods

### Study population and data collection

Snapdragon plants ( *Antirrhinum majus* L., Plantaginaceae) are short-lived, herbaceous perennials. Their geographic distribution is restricted to southern Europe, over the eastern half of Pyrenees Mountains, and extending south and north along the Mediterranean coast from Barcelona to Montpellier ( Khimoun *et al*., 2011) They grow in a variety of environments, including Mediterranean scrubland, scree, understorey vegetation, grassland meadows and sparse shrubland ( Khimoun *et al*., 2013) They are hermaphroditic, self-incompatible, and produce annual inflorescences with zygomorphic flowers pollinated mainly by bumblebees ( *Bombus spp*) and carpenter bees ( *Xylocopa violacea*) and small seeds dispersed by gravity a few meters apart from the plant ( Andalo *et al*., 2010)

Here we focus on snapdragon plants located in a Mediterranean scrubland ecosystem in southern France; between Bages (Latitude: 43.1167; Longitude: 2.9833) and Peyriac de Mer (Latitude: 43.0833; Longitude: 2.9667). Those plants persist on six small isolated rocky hills separated by salt lakes that used to be “Saline d’Estarac”; a site where salt was manufactured from crystalisation ponds using solar evaporation between the years 1007 and 1940 ( Dupont, 1958; Larguier, 2014). As a consequence, this fragmented landscape constrains plants to a patchy distribution.

Between 2010 and 2019, we monitored wild snapdragon plants in the six patches isolated by a few hundred meters of seawater. All patches (numbered 1 to 6 from southwest to northeast) were surveyed between June and early July when plants are sexually mature and the reproductive season ends. All sexually mature snapdragon plants were identified after a thorough search of the area of the six patches (n=12594). Four leaves per plant were sampled for DNA extractions. The geographic location of most plants (12495 out of 12594) was recorded using a GNSS receiver (GNSS device Geo7X, Trimble, Westminster, USA) that provided us with high precision coordinates (sub-meter precision) after the data were post-processed by comparison with data from an independent monitoring station. Based on the barycenter of plant coordinates within a given patch, we calculated the mean distance between patches in meters. We also calculated the mean distances separating plants within patches.

To assess the demography of snapdragon plants, we estimated the annual growth rate in every patch as λ = *N*_*T*–1_/ *N _T_*, where N _T_ is the number of individuals in the current year and N _T-1_ the number of individuals in the previous year. If λ>1 the population is increasing, if λ<1 the population is decreasing, if λ=1 the population is stable ( Pradel, 1996).

### DNA extraction and genotyping

DNA was extracted from leaf tissue using the Nucleospin 96 Plant II (Macherey Nagel, Hoerdt, France). After DNA extraction, samples were amplified at 20 polymorphic microsatellite loci ( Debout *et al*., 2012) using 3 multiplexes (A, B, C, Table 7). Each PCR was performed on a 10 μL total volume: 2 μL of a DNA extract; 3.5 μL QIAGEN Multiplex PCR Kit (Qiagen, Venlo, Limburg, The Netherlands); 0.4 to 0.6 μL of primer mix solution (Eurofins Genomics, Luxembourg, Luxembourg, depending on the multiplex see Table 7) and the remaining volume was completed with DNA-free water. PCRs were performed using a Mastercycler pro Thermal Cyclers (Eppendorf, Hamburg, Germany) with the following protocol for each multiplex: an initial denaturation step at 94 °C for 15 minutes, followed by 35 cycles of 94 °C for 30 seconds; 56°C (primer-specific annealing temperature) for 135 seconds; 72 °C for 30 seconds; and a final extension at 60 °C for 30 minutes. PCR products were sent to the Genoscreen DNA sequencing platform (Lille, France) where samples were analysed on an Automated Capillary DNA Sequencer (ABI 3730, Applied Biosystems, Foster City, CA, USA) using 2 μL of multiplexed PCR products, which were added to 7.75 μL of Hi-Di Formamide and 0.25 μL of the GeneScan-500 LIZ size standard (Applied Biosystems). Allele sizes were scored using GENEIOUS version 9.1 software (Biomatters, Auckland, New Zealand) and double cross-checked. We ensured that there was no genotype duplicates with the R package Allelematch ( Galpern *et al*., 2012). Finally, we ensured marker quality by keeping loci with less than 5% missing data (n=18 microsatellites), and kept individuals with less than 10% missing data (n=12428) for further analysis.

**Table 7.** Primer sequences for 20 microsatellites loci in the *A.majus population*. For more details on the markers see Debout *et al.,* 2012.

*Locus*	*Primer sequences (5’-3’)*	*Multiplex* *markers sets*
*Antibg11*	ATCAACCTGCATCACACCTG TGAATTACGTGAGCGTCGTC	A
*Antibg23*	TCATCACATTTCAATTCATCACA TTGCTTGCTCCAAGTGTTTG	A
*Antibg32*	GATCCGTGAGGAGTGTGGTT CGGCAATCTAATCTCCGAAA	A
*Antibg36*	TGCGTTAGATGATTGCCAAA AAGCTTCCGCTACGTCAGTT	A
*Antibg38*	CCAAGGAGAAGAAAATGTGAGG ATTAGGGAACCTCCAACGCT	A
*Antibg40*	CTCCTCTTCTCACCCGACAT CCCCTCCCTTTCCTAGTTCTT	A
*Antibg02*	TCTGGCAGCAAAAGGTAGAAA CGTGGGAGTTGAAGGAATGA	B
*Antibg03*	TTCTTCAAAGGCAAGCAGGT CATGCTCCTCGTGTGGAAT	B
*Antibg10*	AAACGCATATCCAAAGCAGG CGAAGACCTGCATGACAAAC	B
*Antibg12*	GCATGAAGCCCTGGAAATAA CTCAATGTGACAACTGCATCA	B
*Antibg18*	TTTGCTTTATGTCTTGGTCACCT GACGTGGTGATCAGCTAGGA	B
*Antibg20*	AACCAACAAAGCGAACAACC ATTCGTGACCGTAGAGACCG	B
*Antibg21*	AACTGGGTTTCCTTTCCCAG TTGAGAAATTACCATCATTGTTGTC	B
*Antibg14*	GAGGAAGCGATATCAAGGTATGA TGCTGCCTCCATACAGAAGA	C
*Antibg22*	TTCATCGAATTCTTCGTTCG AAACAACGCAATCCGATCTC	C
*Antibg27*	CGTCGCTAGTTTTCAGCCTC AAATGGTTGCATCCTCCAAG	C
*Antibg29*	TTTGAAAGCATTTTCGGGAC CTGTACACTCTGCCGGTCAA	C
*Antibg30*	TCCTTTCATTCCTCTCCATCA TTTGGAGCCACCTTCATTTC	C
*Antibg33*	CAAATGACATCCAAAAGATAATACAA AGAGATTTAGGCGATACAAGCA	C
*Antibg39*	ATACTGGGACCCACAAGCTG CTTCACCAAACCGCAAGATT	C

### Population genetic differentiation

We estimated the genetic diversity of snapdragon plants in the six patches by using Nei’s multilocus estimates of expected heterozygosity (Hs), polymorphic information content (PIC), and allelic richness (A). Analyses were conducted with the FRANz software ( Riester *et al*., 2009; Riester *et al*., 2010).

We estimated genetic differentiation between pairs of patches by calculating Wright's fixation indices (Fst) using Weir and Cockerham's θST estimates (1984) in GENODIVE v2.0 ( Meirmans & Tienderen, 2004). We also estimated global Fst amongst patches. Genetic differentiation was also measured per year to investigate temporal variation in Fst.

### Parentage analysis and pedigree reconstruction

We ensured that the set of microsatellite markers that we used was highly reliable for parentage analysis by estimating Parent-Pair exclusion probability (PPexp, Wang, 2007). PPexp for each marker and cumulative PPexp were estimated with the FRANz software ( Riester *et al*., 2009; Riester *et al*., 2010). Individuals were assigned to parental genotypes using a Bayesian pedigree reconstruction approach that takes into account uncertainty about age; with age being estimated by the software on the basis the first year of sampling of each plant. We performed the parentage analysis in FRANz with the default parameter settings, except for: maximum number of candidate fathers (Nmax=14000), age range in which females and males can reproduce (femrepro=0:20; malerepro=0:20) to largely cover their lifespan, minimum number of typed loci (mintyped=16), convergence tolerance (saepsilon=0.1) and increment in the steady states distribution variation (sadelta=0.01) (see Almudevar, 2003 for more details on parameterisation).

We only included in the final pedigree the parentage assignments with a posterior probability higher than 0.95, which indicates that this parentage link was found in at least 95 of the 100 potential pedigrees reconstructed by using the assignment approach presented above. We only kept individuals with two reliably assigned parents (triads) to avoid false assignments arising from overlapping generations when using dyads. Each triad (offspring, parent1 & parent2) is also characterised by location information (patches from 1 to 6), GNSS coordinates, and their first year of sampling. We produced descriptive pedigree statistics (e.g. number of parent-offspring relationships, number of full sibling links, etc.) by using the Sequoia (v2.1.3, updates for hermaphrodites, Huisman, 2017) and Pedantics ( Morrissey & Wilson, 2010) R packages. The type of family relationship between individuals were identified with the function GetRelCat of the Sequoia package. Individuals were then assigned to families to identify the different types of families, the number of family members, and their spread across generations with the package kinship2 ( Sinnwell *et al*., 2014) (makefamid function).

### Functional connectivity and geographical distance among patches

Based on the parentage assignment and the location of plants, we estimated the number of within-patch dispersal events (i.e., when parents and offspring are found in the same patch), and between-patch dispersal events (i.e., when at least one parent is found on a different patch than the offspring). Dispersal distance was estimated by the geographical Euclidean distance in meters between parents and offspring using the R package Raster ( Hijmans *et al*., 2021). The closest parent (P1) was consistently located in the same patch than the offspring, which was expected because seeds are dispersed by gravity. Functional connectivity was estimated as the ratio of the total number of between-patch dispersal events and the total number of effective dispersal events overall patches. We also estimated per patch, outbound connectivity as the percentage of offspring on a different patch with the farthest parent on a given focal patch, inbound connectivity as the percentage of offspring on the given focal patch with the farthest parent on a different patch and total connectivity per patch as the sum of inbound and outbound connectivity. Finally, we tested whether patch-pairwise functional connectivity – estimated as the sum of effective dispersal events between two patches divided by the sum of effective dispersal events of these patches multiplied by 100 – was correlated with between-patch geographical distance by using a Spearman correlation test.

### Reproductive success

To assess if functional connectivity was effective, we used the multigenerational pedigree to calculate the reproductive success estimated by the number of offspring of each plant resulting from a between-patch dispersal event, and each plant with resident parents. We removed plants sampled during the last two years of the survey (2018 and 2019) to avoid a temporal bias because the probability of finding their offspring in the field was weak. We assessed if the reproductive success was different between plants resulting from between-patch dispersal events, and plants with resident parents. To this aim, we built a negative binomial linear mixed model with a logit function to linearize the reproductive success count data, which accounts for count data overdispersion. We included the “dispersal status” of parents as a fixed effect (0 for resident parents and 1 for parents from different patches). We also included in the model the random effects on the intercept of the identity of patches and the closest and farthest parent to account for the non-independence of observations due to their location and genotype. This model was computed with the glmmTMB package ( Magnusson *et al*., 2021). We checked that the necessary assumptions of the model were respected (e.g., uniformity, overdispersion, outliers) with the DHARMA package ( Hartig & Lohse, 2021). All analyses have been conducted with R version 3.6.3 ( R Core Team, 2020).

## Data availability

### Underlying data

Zenodo: Code and data for "Wild snapdragon plant pedigree sheds light on limited connectivity enhanced by higher migrant reproductive success in a fragmented landscape",

https://doi.org/10.5281/zenodo.5682659 ( Gervais *et al*., 2021)

This project contains the following underlying data:

- freq_MCMC_1
- locisummary_MCMC_1.txt
- mcmc_MCMC_1.log
- mismatch_MCMC_1.txt
- output.txt
- parentage_MCMC_1.csv
- pedigree_MCMC_1.dat
- pedigree_MCMC_1.txt
- simulation_MCMC_1.txt
- summary_MCMC_1.txt
- geno_20102019.dat
- pedigree&location_metadata.txt
- pedigree_tryads_P095.Rdata

Data are available under the terms of the Creative Commons Attribution 4.0 International license (CC-BY 4.0).
