# Supplementary material for: Wild snapdragon plant pedigree sheds light on limited connectivity enhanced by higher migrant reproductive success in a fragmented landscape
Source: Open Res Eur. 2023 Jan 12;1:145. Originally published 2021 Dec 1. [Version 2] doi: 10.12688/openreseurope.14281.2 (PMC10446054; doi:10.12688/openreseurope.14281.2)
Supplement: Peer review history

# Peer review: Reviewer response for version 2

**DOI:** 10.21956/openreseurope.16711.r30618

**Published:** 2023-02-16

**Authors:** Yann X. C. Bourgeois

**Affiliations:** 1 School of Biological Sciences, University of Portsmouth, Portsmouth, UK

The authors have answered all my comments. I understand that some aspects may be further investigated in future studies. I do not have any major concerns about this work.

 All the best.

Is the study design appropriate and does the work have academic merit?

Yes

Is the work clearly and accurately presented and does it cite the current literature?

Yes

If applicable, is the statistical analysis and its interpretation appropriate?

Partly

Are all the source data underlying the results available to ensure full reproducibility?

Yes

Are the conclusions drawn adequately supported by the results?

Partly

Are sufficient details of methods and analysis provided to allow replication by others?

Yes

Reviewer Expertise:

I am an evoutionary biologist interested in population genetics and genomics, with a focus on fast adaptation and transposable elements.

I confirm that I have read this submission and believe that I have an appropriate level of expertise to confirm that it is of an acceptable scientific standard.

# Peer review: Reviewer response for version 2

**DOI:** 10.21956/openreseurope.16711.r30617

**Published:** 2023-01-13

**Authors:** Josselin Clo

**Affiliations:** 1 Charles University, Prague, Czech Republic

I am happy with the authors' responses and modifications to the manuscript.

 I found the text much clearer after taking into account the reviewers' comments, and I am still thinking it is a nice article and contribution to the field.

Is the study design appropriate and does the work have academic merit?

Yes

Is the work clearly and accurately presented and does it cite the current literature?

Yes

If applicable, is the statistical analysis and its interpretation appropriate?

Yes

Are all the source data underlying the results available to ensure full reproducibility?

Yes

Are the conclusions drawn adequately supported by the results?

Yes

Are sufficient details of methods and analysis provided to allow replication by others?

Partly

Reviewer Expertise:

I am an evolutionary biologist exploring how key life-history traits (inbreeding and polyploidy) affect the evolutionary potential of populations.

I confirm that I have read this submission and believe that I have an appropriate level of expertise to confirm that it is of an acceptable scientific standard.

# Peer review: Reviewer response for version 1

**DOI:** 10.21956/openreseurope.15406.r29502

**Published:** 2022-07-07

**Authors:** Yann X. C. Bourgeois

**Affiliations:** 1 School of Biological Sciences, University of Portsmouth, Portsmouth, UK

In this work, the authors contrast functional and effective connectivity at a small spatial scale in a population of wild snapdragons divided into six distinct patches. They conducted a demographic survey over several years and estimated migration rates between isolated patches to test whether higher reproductive success of migrants may counterbalance genetic isolation. They show that despite barriers to dispersal between patches, the higher fitness of individuals with parents from different patches may compensate for their relative rarity and rescue populations. The analyses are sound and the amount of fieldwork this study represents is impressive. The manuscript is well written.

 After reading the Abstract and Introduction, and references therein, I was left with the impression that the authors think that populations may **benefit** from a fragmented habitat here. While it is clear that populations do not go extinct, they may still be growing at a lower rate than more connected populations. Instead of benefitting populations, fragmentation increases inbreeding within patches, lowering genetic diversity, while migration rescues local patches, which can keep growing. Without a proper comparison of this population with another large, non-fragmented one, it remains difficult to draw any clear conclusion about the meaning of positive growth rates. I feel like this should be more clearly stated.

**Comments about functional connectivity estimates:**

- Pedigrees are useful, but relying solely on them leaves the door open to ascertainment biases due to missing parents/offsprings or unassigned individuals. This means that the “2.9% of offspring [that] had one parent on a different patch” is probably an underestimate of the actual connectivity. Ghost (unsampled) populations may also contribute to the homogenization of gene pools, and it would be worth presenting more clearly how many individuals had one or two unidentified parents in each patch. I would not be surprised if that number was higher in the patches bordering the study area (i.e. 1 and 6).
- I do not agree that functional connectivity is “weak”. FST are significant but low (ranging from 0.01 to 0.08 at most). Using the Wright’s equation as a rule of thumb (FST ≈ 1/(4Nem + 1)), this suggests a number of migrant alleles between patches that is at the very least around 3 per generation. This is higher than the classical "One migrant per generation" rule used to determine whether populations are drifting apart. This warrants a more nuanced discussion throughout the paper.
- Other methods may also be used to estimate relatedness for all pairs of geolocated individuals, between and within patches. The authors could then assess whether there is any significant barrier to gene flow. One example of spatially-explicit method that can handle microsatellites is EEMS (Petkova, Novembre, & Stephens, 2015 ^1^). It might be worth applying it here to examine whether the diffusion of alleles is significantly higher between patches than within them. Such analyses could include the vast majority of individuals not included in the pedigree.

**Comments on effective connectivity (migrant reproductive success):**

- There are many well documented examples showing an advantage for migration that restores genetic diversity and, e.g., limits the exposure of deleterious alleles at the homozygous state. One of the most interesting aspects of the study in my opinion is the quantitative estimate of migrant reproductive success. It may be worth providing an estimate of individual inbreeding to test for a correlation between reproductive success and heterozygosity for example. One could also compare populations growth rates with the number of migrants detected every year, or with average FST.

**Miscellaneous:**

- Table 3: I assume these statistics are produced by pooling all individuals. I would recommend an analysis per year and per population. Deviations from Hardy-Weinberg equilibrium may reveal strong local inbreeding for example. In addition, the lack of deviation from Hardy-Weinberg equilibrium suggests that Wahlund effects due to underlying population structure are not pronounced. Some authors would even consider this as a lack of evidence for any significant population structure (cf comment above about the “weak” connectivity).

I hope you find these comments constructive.

Is the study design appropriate and does the work have academic merit?

Yes

Is the work clearly and accurately presented and does it cite the current literature?

Yes

If applicable, is the statistical analysis and its interpretation appropriate?

Partly

Are all the source data underlying the results available to ensure full reproducibility?

Yes

Are the conclusions drawn adequately supported by the results?

Partly

Are sufficient details of methods and analysis provided to allow replication by others?

Yes

Reviewer Expertise:

I am an evoutionary biologist interested in population genetics and genomics, with a focus on fast adaptation and transposable elements.

I confirm that I have read this submission and believe that I have an appropriate level of expertise to confirm that it is of an acceptable scientific standard, however I have significant reservations, as outlined above.

## References

[1] : Visualizing spatial population structure with estimated effective migration surfaces. *Nat Genet* .2016;48(1) : 10.1038/ng.3464 94-100 10.1038/ng.3464 26642242PMC4696895

# Response

**Published:** 2022-12-14

**Authors:** Laura Gervais

**Affiliations:** CNRS, France

We thank you for your comments that helped improving this paper and gave us perspective for future research questions on this study system. You will find our point by point response preceded by “>>Authors response". All line numbers refer to the updated manuscript file containing changes highlighted in yellow. Please note that the track-changed manuscript is available on zenodo here https://doi.org/10.5281/zenodo.7438502. ----------------------------------------------------------------

 Yann X. C. Bourgeois, School of Biological Sciences, University of Portsmouth, Portsmouth, UK

 In this work, the authors contrast functional and effective connectivity at a small spatial scale in a population of wild snapdragons divided into six distinct patches. They conducted a demographic survey over several years and estimated migration rates between isolated patches to test whether higher reproductive success of migrants may counterbalance genetic isolation. They show that despite barriers to dispersal between patches, the higher fitness of individuals with parents from different patches may compensate for their relative rarity and rescue populations. The analyses are sound and the amount of fieldwork this study represents is impressive. The manuscript is well written. After reading the Abstract and Introduction, and references therein, I was left with the impression that the authors think that populations may benefit from a fragmented habitat here. While it is clear that populations do not go extinct, they may still be growing at a lower rate than more connected populations. Instead of benefitting populations, fragmentation increases inbreeding within patches, lowering genetic diversity, while migration rescues local patches, which can keep growing. Without a proper comparison of this population with another large, non-fragmented one, it remains difficult to draw any clear conclusion about the meaning of positive growth rates. I feel like this should be more clearly stated.

**>> Author response:** We thank the reviewer for pointing this issue which was not our intention. We clarified the text throughout the paper, and in particular in the introduction, to outline that we are aware of the negative impact of fragmentation, and that we do not think snapdragon plant populations benefit directly from fragmentation. We agree with you. Our aim is to outline that based on the literature, mechanisms at play in populations that are surviving and expanding in fragmented landscapes are unclear. We do not intend to interpret positive growth rates as a property of fragmented versus non-fragmented large populations. We simply present here an example of limited functional connectivity, which role played against reproductive isolation may have been underestimated if we had not found out that the few propagule exchanges were efficient as they were associated with higher reproductive success.

 Comments about functional connectivity estimates:

- Pedigrees are useful, but relying solely on them leaves the door open to ascertainment biases due to missing parents/offsprings or unassigned individuals. This means that the “2.9% of offspring [that] had one parent on a different patch” is probably an underestimate of the actual connectivity. Ghost (unsampled) populations may also contribute to the homogenization of gene pools, and it would be worth presenting more clearly how many individuals had one or two unidentified parents in each patch. I would not be surprised if that number was higher in the patches bordering the study area (i.e. 1 and 6).

**>> Author response: **We agree that true estimates of connectivity can only be higher than estimates obtained from pedigrees because of the presence of ghost parents. This is a widely acknowledged limitation of all pedigree-based connectivity studies. There are two ways to overcome at least partly this issue: 1. focus on robust “family triangles” (two parents + offspring) across patches that were exhaustively surveyed (with of course a few missing samples). 2. use geographic sites which natural setting is similar to an optimal experimental design (eg. when 6 patches of plants are located on islands, or 3 lagoons around an island are far from the main coast). As a result, estimated connectivity can be used to infer migration processes between surveyed patches because a large sample of plants is used but these connectivity estimates cannot be used to infer what is ongoing beyond this geographic scale. Here, the pedigree analysis allowed us to identify directional trends in connectivity, which allowed us to identify the plants directly involved in effective connectivity events between the 6 sites that are surveyed. We had already mentioned in the text that the presence of ghost parents "suggests migration from outside the studied area” the text. We accommodated your comment in the revised text by presenting numbers for unidentified parents L111, L125-128.

- I do not agree that functional connectivity is “weak”. FST are significant but low (ranging from 0.01 to 0.08 at most). Using the Wright’s equation as a rule of thumb (FST ≈ 1/(4Nem + 1)), this suggests a number of migrant alleles between patches that is at the very least around 3 per generation. This is higher than the classical "One migrant per generation" rule used to determine whether populations are drifting apart. This warrants a more nuanced discussion throughout the paper.

**>> Author response:** We agree with the reviewer that with a few migrants per generation (not far from the “one migrant per generation rule”), connectivity plays an important role. This is empirically supported by our estimates of connectivity, and this is our take home message. We now mention more clearly this aspect in the text and present scenarii of migration counterbalancing drift rooted in our coherent FST and connectivity results L185-190; L195-196. We also removed “weak connectivity” from the text and nuanced the discussion of this aspect throughout the paper.

- Other methods may also be used to estimate relatedness for all pairs of geolocated individuals, between and within patches. The authors could then assess whether there is any significant barrier to gene flow. One example of spatially-explicit method that can handle microsatellites is EEMS (Petkova, Novembre, & Stephens, 20151). It might be worth applying it here to examine whether the diffusion of alleles is significantly higher between patches than within them. Such analyses could include the vast majority of individuals not included in the pedigree.

**>> Author response:** We agree that other relatedness methods using all pairs of individuals can also be used to infer spatial patterns of effective migration. Here, the pedigree analysis allowed us to identify directional trends in connectivity, notably based on robust “family triangles” (two parents + offspring), which allowed us to identify the plants directly involved in effective connectivity events between the 6 sites that are surveyed. This pedigree approach has the advantage of estimating both migration events and reproductive success. As outlined in the text, we want to encourage studies estimating connectivity to also infer reproductive success which is for now, to our knowledge, only possible with a pedigree approach. We thank you for outlining the interest of using the EEMS approach and are enthusiastic about using it in another study more specifically aiming at evaluating the role of ecological barriers.

 Comments on effective connectivity (migrant reproductive success):

- There are many well documented examples showing an advantage for migration that restores genetic diversity and, e.g., limits the exposure of deleterious alleles at the homozygous state. One of the most interesting aspects of the study in my opinion is the quantitative estimate of migrant reproductive success. It may be worth providing an estimate of individual inbreeding to test for a correlation between reproductive success and heterozygosity for example.

**>> Author response:** We agree that the link between migration and a higher reproductive success is a key finding of our study and that research aiming at understanding the underlying mechanisms is very promising. We also agree that HFC (heterozygote-fitness correlations) could be used to investigate the link between inbreeding and reproductive success, which may be itself linked to migration. However, many ecological or genetic mechanisms may be at play here. An investigation of the inbreeding-fitness correlation would be in itself a demanding study requiring to explore many evolutionary scenarii. As such, it would be difficult to include this aspect in this paper without carrying the reader away from the take home message of this study. We thank the reviewer for outlining this research track that we will hope to follow in the near future.

- One could also compare populations growth rates with the number of migrants detected every year, or with average FST.

**>> Author response:** We agree that behind the test proposed by the reviewer lies a very interesting research question. However, we do not think that our dataset allows to reliably test for this hypothesis. There is likely a time lag between the current arrival of migrants and their participation to the population growth, and a temporal disconnection with genetic differentiation. We also think that average FST estimates may not be very well fitted for this type of statistical analysis. We took this comment into account by outlining in the revised text that it would be interesting to investigate the link between the growth rate and differentiation (or in fact connectivity) but did not go further in the details to avoid speculation L227-228; L230-232.

 Miscellaneous:

- Table 3: I assume these statistics are produced by pooling all individuals. I would recommend an analysis per year and per population.Deviations from Hardy-Weinberg equilibrium may reveal strong local inbreeding for example. In addition, the lack of deviation from Hardy-Weinberg equilibrium suggests that Wahlund effects due to underlying population structure are not pronounced. Some authors would even consider this as a lack of evidence for any significant population structure (cf comment above about the “weak” connectivity).

**>> Author response:** Indeed, the statistics of table 3 are made by pooling individuals among years and patches. We added this information in the legend of table 3 L86-87. In the genetic differentiation analysis, we only used markers that were at HW equilibrium as required to respect statistical assumptions. We added this information in the methods section L306-308. Since FST was very low, there is little opportunity for Wahlund effects. Using deviation from HW equilibrium to reveal the lack of between-population differentiation in these snapdragon patches would be unreliable. I hope you find these comments constructive.

# Peer review: Reviewer response for version 1

**DOI:** 10.21956/openreseurope.15406.r28623

**Published:** 2022-02-21

**Authors:** Josselin Clo

**Affiliations:** 1 Charles University, Prague, Czech Republic

I had the pleasure to read and review the manuscript “Wild snapdragon plant pedigree shed lights on limited connectivity enhanced by higher migrant reproductive success in a fragmented landscape”, from Gervais *et al*. The aim of the manuscript is to investigate the Snapdragon demographic dynamic during several years, in a population fragmented in several isolated patches. They associated the demographic survey with an analysis of the effective connectivity between patches in order to understand the demographic results. The main results of the study are that all the patches within the populations are increasing their demographic sizes, despite very low gene flows among patches. Nevertheless, if the genetic exchanges are rare, the fitness of migrant plants is higher than the one of local plants, potentially explaining the demographic patterns.

 Overall, the manuscript is well-written. The dataset used is impressive, with a sampling of more than 12.000 plants over ten years. The introduction prepares the reader for what will be presented and discussed later in the manuscript. It offers a quick but comprehensive summary of the effect of habitat fragmentation on the evolutionary potential of the (sub-)populations, and how the (effective) connectivity between fragmented habitats can help the populations in overcoming the above-mentioned negative effects. The other sections are also overall well written, in a concise style which is nice to read. The results answered the questions presented in the introduction. The results are not overstated. In addition, the data and Rcode used are already available in public repositories.

 I have only one major comment that I would like to discuss with the authors, and several “cosmetic” minor comments, that I hope will help the authors in increasing a little bit more the quality and clarity of the manuscript.

**Major comment:**

****

 The authors stated that the observed population expansion is due to the higher reproductive success after migration, which compensates for the other deleterious effects of habitat fragmentation. It could be true, but as presented, it is not the only possible explanation. The high number of sampling plants suggests that the population of Snapdragon was already well established in this former industrial location, it is possible that the population is just well adapted to its environment, and that even without the help of the fittest migrants the sub-populations would be in a demographic expansion?

 I think that you could try to correlate the growth rates of patches with your measures of connectivity (the functional and effective connectivity), to see if higher growth rates are associated with more fittest migrants arriving in the patches?

****

**Minor comments:**

****

 The formatting of the manuscript avoids giving the line numbers. I tried to be as clear as possible for you to find the location of the comment, by advance sorry if it is not clear.

****

**Introduction:**

****

 First paragraph: the authors can also mention the Wood *et al.* (2016 ^1^) meta-analysis showing that smaller demographic population sizes are not associated with a decrease in adaptive potential.

**Results:**

*Demographic expansion:*

 Table 2: The legend indicates that a growth rate higher than 1 should be in bold, but this is not the case. Either modify the legend or the table. Some growth rates are incredibly high (for example patch 1 in 2015), is it a real growth rate, or did you forget the brackets?

*Genetic diversity:*

 The material and method section being after the result section, some parameters and their abbreviations are not described. Please indicate what is Hs the first time you mention it, and what represents the variation around the mean value (95% confidence interval, standard deviation?), and indicate it also in the M&M section.

*Low functional connectivity between the six patches:*

 Figure 4: The visual quality of figure 4 is low compared to the three others, is it possible to improve it?

**Discussion:**

****

 First paragraph: Please propose alternative scenarios.

******

*Higher reproductive success associated with connectivity:*

 Please give more details of why migrant plants can have higher fitness than the local ones.

**

**Material and methods:**

****

 GPS coordinates: Most of the points were recorded with the GNSS receiver, but how were the few hundred remaining points recorded?

 Statistical tests: Several p-values are reported in the “Results section” (for Fst estimations for example), but the statistical tests are not described in the material and method section. Please indicate the test you performed through the whole M&M section.

 Parentage analysis: Please indicate the default values of other parameters, if there are not too many.

Is the study design appropriate and does the work have academic merit?

Yes

Is the work clearly and accurately presented and does it cite the current literature?

Yes

If applicable, is the statistical analysis and its interpretation appropriate?

Yes

Are all the source data underlying the results available to ensure full reproducibility?

Yes

Are the conclusions drawn adequately supported by the results?

Yes

Are sufficient details of methods and analysis provided to allow replication by others?

Partly

Reviewer Expertise:

I am an evolutionary biologist exploring how key life-history traits (inbreeding and polyploidy) affect the evolutionary potential of populations.

I confirm that I have read this submission and believe that I have an appropriate level of expertise to confirm that it is of an acceptable scientific standard, however I have significant reservations, as outlined above.

## References

[1] : Are heritability and selection related to population size in nature? Meta-analysis and conservation implications. *Evol Appl* .9(5) : 10.1111/eva.12375 640-57 10.1111/eva.12375 27247616PMC4869407

# Response

**Published:** 2022-12-14

**Authors:** Laura Gervais

**Affiliations:** CNRS, France

We would like to thank the reviewer for his positive feedbacks and constructive comments. You will find our point by point response preceded by “>>Authors response". All line numbers refer to the updated manuscript file containing changes highlighted in yellow. Please note that the track-changed manuscript is available on zenodo here https://doi.org/10.5281/zenodo.7438502.

 --------------------------------------------------------------------------------------

 I had the pleasure to read and review the manuscript “Wild snapdragon plant pedigree shed lights on limited connectivity enhanced by higher migrant reproductive success in a fragmented landscape”, from Gervais et al. The aim of the manuscript is to investigate the Snapdragon demographic dynamic during several years, in a population fragmented in several isolated patches. They associated the demographic survey with an analysis of the effective connectivity between patches in order to understand the demographic results. The main results of the study are that all the patches within the populations are increasing their demographic sizes, despite very low gene flows among patches. Nevertheless, if the genetic exchanges are rare, the fitness of migrant plants is higher than the one of local plants, potentially explaining the demographic patterns. Overall, the manuscript is well-written. The dataset used is impressive, with a sampling of more than 12.000 plants over ten years. The introduction prepares the reader for what will be presented and discussed later in the manuscript. It offers a quick but comprehensive summary of the effect of habitat fragmentation on the evolutionary potential of the (sub-)populations, and how the (effective) connectivity between fragmented habitats can help the populations in overcoming the above-mentioned negative effects. The other sections are also overall well written, in a concise style which is nice to read. The results answered the questions presented in the introduction. The results are not overstated. In addition, the data and Rcode used are already available in public repositories. I have only one major comment that I would like to discuss with the authors, and several “cosmetic” minor comments, that I hope will help the authors in increasing a little bit more the quality and clarity of the manuscript. The authors stated that the observed population expansion is due to the higher reproductive success after migration, which compensates for the other deleterious effects of habitat fragmentation. It could be true, but as presented, it is not the only possible explanation. The high number of sampling plants suggests that the population of Snapdragon was already well established in this former industrial location, it is possible that the population is just well adapted to its environment, and that even without the help of the fittest migrants the sub-populations would be in a demographic expansion?

**>> Author response:** We fully agree that one reason behind the expansion of these plants is likely that they are well adapted to their current environment. We did not wish to imply that expansion was caused by the higher reproductive success of migrants, and we did not wish to exclude other mechanisms. We only meant to imply that in a context of limited propagule exchanges (functional connectivity was low), a higher reproductive success after migration increases the chances that migrants participate to population dynamics and counteracts reproductive isolation between patches. We thank the reviewer for pointing this out. We have clarified this aspect throughout the text. I think that you could try to correlate the growth rates of patches with your measures of connectivity (the functional and effective connectivity), to see if higher growth rates are associated with more fittest migrants arriving in the patches?

**>> Author response:** Thank you for pointing out this interesting hypothesis. We included some text to mention that it would be interesting to investigate the link between the growth rate of patches and their connectivity L228-229. However, we do not think it is possible to test this hypothesis using our dataset L222-225. There is likely a time lag between the arrival of migrants and their participation to a given patch growth rate in wild snapdragon populations. We also think that it would be difficult to interpret a correlation between higher growth rates and the arrival of fitter migrants because this correlation would not imply causality in the wild because of multiple other drivers might be involved.

 The formatting of the manuscript avoids giving the line numbers. I tried to be as clear as possible for you to find the location of the comment, by advance sorry if it is not clear.

**>> Author response:** Likewise. We had included line numbers but ORE removed them, which we think is deleterious to the review process for both authors and reviewers. We uploaded a revised version of the paper that includes line numbers to which we refer to in this response letter. If these numbers were again to be removed from the main document, know that we uploaded on the zenodo server a version named “track changes” that includes line numbers and where text modifications are outlined.

 Introduction:

 First paragraph: the authors can also mention the Wood et al. (20161) meta-analysis showing that smaller demographic population sizes are not associated with a decrease in adaptive potential.

**>> Author response: **We thank the reviewer for this reference. Yet we think this reference is not adequate for our paper. Wood et al showed no reduction of narrow-sense heritability with decreasing population size. Beyond the fact that choosing h² to compare the adaptive potential of populations from different contexts raises several issues, this study is not directly related to connectivity and fragmentation.

 Results:

 Demographic expansion:

 Table 2: The legend indicates that a growth rate higher than 1 should be in bold, but this is not the case. Either modify the legend or the table. Some growth rates are incredibly high (for example patch 1 in 2015), is it a real growth rate, or did you forget the brackets?

**>> Author response: **Thanks for pointing this out. We clarified the legend and the contents of the table to outline numbers that are not growth rates but represent population sample sizes L65-69 . We did put population sample sizes between brackets to indicate when their short time fluctuations in patches characterized by small population sample sizes would result in abnormally high growth rates (e.g., from 1 to 50 plants over a year would lead to a growth rate value of 50). After discussing and revisiting the sampling data, we decided that patch 1 growth rates should be considered with extreme caution and specified this aspect in the text (Legend of Table 2 and methods, L67-69; L268-272). This is because the spatial heterogeneity of patch 1 might have compromised the possibility to replicate the exact same sampling area from one year to the next (notably in 2015). These short-term fluctuations do not challenge our conclusion about the global increase in population size. Nevertheless, we consider that these fluctuations limit our perspective on growth rates and exclude the possibility of using a correlative approach to analyse their variation beyond evaluating global trends. Genetic diversity:

 The material and method section being after the result section, some parameters and their abbreviations are not described. Please indicate what is Hs the first time you mention it, and what represents the variation around the mean value (95% confidence interval, standard deviation?), and indicate it also in the M&M section.

**>> Author response:** Done L72, L299-300.

 Low functional connectivity between the six patches:

 Figure 4: The visual quality of figure 4 is low compared to the three others, is it possible to improve it?

**>> Author response:** Done.

 Discussion:

 First paragraph: Please propose alternative scenarios.

**>> Author response:** We followed the reviewer’s suggestion and clarified the two scenarios that both rely on the balance between drift and migration. One scenario relies on the equilibrium of these forces, with a history of drift and likely low migration resulting in genetic differentiation and the other scenario involves a past history of divergence (linked to the same drifting mechanisms) but in the process of being resolved by connectivity. Connectivity might be recent and results at least partly from the demographic expansion that would have increased pollen flow. We did not enter into that much details in the text because we do not want to speculate too much. These two scenarii are now presented in the revised text L182-187. Higher reproductive success associated with connectivity:

 Please give more details of why migrant plants can have higher fitness than the local ones.

**>> Author response:** We followed the reviewer’s comment by adding more details L217. In terms of phenotypic performance, we now mention in the text the example from Williams et al 2016 who said “Plant height and dispersal ability evolved more rapidly in patchier experimental landscapes, suggesting that fragmentation can select for more rapid invasion velocity“. We also added text about an alternative hypothesis, which is that the higher reproductive success of migrants can be explained by random processes (e.g. colonisation waves)L213-214. Obviously, we did not enter in too many details in the text of the paper because we do not want to speculate too much. In order to correctly investigate these mechanisms, it would be necessary to conduct a different type of study to test for these hypotheses. Material and methods:

 GPS coordinates: Most of the points were recorded with the GNSS receiver, but how were the few hundred remaining points recorded?

**>> Author response**: Indeed 99 individuals did not have geographic coordinates, but have a sampling location noted as patch 1,2,3,4,5,6, which is sufficient for the connectivity measures. We add this information L263-264.

 Statistical tests: Several p-values are reported in the “Results section” (for Fst estimations for example), but the statistical tests are not described in the material and method section. Please indicate the test you performed through the whole M&M section.

**>> Author response:** Done L307-308. Parentage analysis: Please indicate the default values of other parameters, if there are not too many.

**>> Author response: **There are over 40 different arguments in the FRANz function that are set to the default value. We think it is better to specify only those not set to default values in the text and invite readers to refer to the Franz manual on the Franz github repository to access all the default values https://github.com/lima1/franzpedigree.
